# Regenerative potential of basic fibroblast growth factor contained in biodegradable gelatin hydrogel microspheres applied following vocal fold injury: Early effect on tissue repair in a rabbit model

**DOI:** 10.1016/j.bjorl.2019.09.003

**Published:** 2019-10-18

**Authors:** Mitsuyoshi Imaizumi, Ryosuke Nakamura, Yuta Nakaegawa, Bayu Tirta Dirja, Yasuhiro Tada, Akiko Tani, Takashi Sugino, Yasuhiko Tabata, Koichi Omori

**Affiliations:** a Fukushima Medical University, School of Medicine, Department of Otolaryngology, Fukushima, Japan; b Kyoto University, Department of Otolaryngology, Kyoto, Japan; c Shizuoka Cancer Center, Division of Pathology, Shizuoka, Japan; d Kyoto University, Institute for Frontier Life and Medical Sciences, Department of Regeneration Science and Engineering, Laboratory of Biomaterials, Kyoto, Japan

**Keywords:** Vocal fold injury, Biodegradable gelatin hydrogel microspheres, Basic fibroblast growth factor (bFGF, FGF-2)

## Abstract

**Introduction:**

Postoperative dysphonia is mostly caused by vocal fold scarring, and careful management of vocal fold surgery has been reported to reduce the risk of scar formation. However, depending on the vocal fold injury, treatment of postoperative dysphonia can be challenging.

**Objective:**

The goal of the current study was to develop a novel prophylactic regenerative approach for the treatment of injured vocal folds after surgery, using biodegradable gelatin hydrogel microspheres as a drug delivery system for basic fibroblast growth factor.

**Methods:**

Videoendoscopic laryngeal surgery was performed to create vocal fold injury in 14 rabbits. Immediately following this procedure, biodegradable gelatin hydrogel microspheres with basic fibroblast growth factor were injected in the vocal fold. Two weeks after injection, larynges were excised for evaluation of vocal fold histology and mucosal movement.

**Results:**

The presence of poor vibratory function was confirmed in the injured vocal folds. Histology and digital image analysis demonstrated that the injured vocal folds injected with gelatin hydrogel microspheres with basic fibroblast growth factor showed less scar formation, compared to the injured vocal folds injected with gelatin hydrogel microspheres only, or those without any injection.

**Conclusion:**

A prophylactic injection of basic fibroblast growth factor -containing biodegradable gelatin hydrogel microspheres demonstrates a regenerative potential for injured vocal folds in a rabbit model.

## Introduction

Treatment of dysphonia after vocal fold injuries secondary to surgery, trauma and inflammation post infection remains a challenge,^1^ and reports suggest that postoperative dysphonia is mostly caused by vocal fold scarring.^2^ Dailey and Ford reported that some of the most common causes of the disorder are likely to be postsurgical and iatrogenic.^3^ Benninger et al. reported that careful preoperative, operative and postoperative management of vocal fold surgery can reduce the risk of scar formation.^4^ However, depending on the extent and depth of the vocal fold injury, scar prevention can be intractable in the natural healing process.^5^

Numerous studies on the treatment of vocal fold scarring, including animal experimentation^6^, ^7^ and clinical applications,^8^, ^9^ have been performed to solve the problem of intractable scar formation; however, a consensus for the effective treatment of scar formation has yet to be reached. The treatment approaches for vocal fold scarring can have a certain positive effect, although its regenerative capacity might be insufficient to completely replace chronic vocal fold scarring. Therefore, novel regenerative approaches to treat vocal fold scarring are required.

One potential approach for vocal fold scarring associated with surgery is scar formation recovery using regenerative medicine strategies, which focus on the regeneration of human cells, tissues or organs to restore impaired function. Regenerative strategies include the use of growth factors, cells, and tissue scaffolds,^10^ and one of the most expected regenerative approaches is cell transplantation. Various cell sources have been used for treating vocal fold scarring in order to elucidate the mechanism of vocal fold repair. Several previous studies have reported fibroblasts,^11^ mesenchymal stem cells,^12^ adipose-derived stem cells,^13^ embryonic stem cells^14^ and induced pluripotent stem cells^15^ to have a positive effect on vocal fold scarring. However, clinical cell transplantation requires strict safety management and is expensive. Furthermore, the possibility of transplant rejection has limited use for tissue-engineering applications in humans. An alternative approach for treating scarred vocal folds is the application of a combination of growth factor and scaffold. Previous in vivo studies have reported the therapeutic potential of a combination of hepatocyte growth factor hydrogel and collagen-gelatin scaffold with Basic Fibroblast Growth Factor (bFGF); however, complete restoration could not be achieved.^16^, ^17^ Complete recovery of matured, chronic vocal fold scarring associated with surgery is therefore challenging.

Another possible solution for vocal fold scarring associated with surgery is a preventative approach. The effectiveness of vocal fold scarring prevention by local application of saline with bFGF has been previously demonstrated;^18^ however, in that study, the injection was performed before the surgical procedure to reduce the risk of leakage from the injured area. Theoretically, bFGF can potentially increase tumor vascularity, blood flow and growth.^19^ Therefore, to minimize the unnecessary risk of tumor growth, it is desirable to determine the application of bFGF injection not before the surgical procedure, but after observing the extent and depth of the vocal fold damage. In addition, as bFGF is a growth factor with a short half-life,^8^, ^20^ multiple injections are required to obtain the regenerative effects.^21^ In the current study, gelatin hydrogel microspheres, of which viscosity is different from that of saline, with bFGF were selected in order to reduce leakage, and to use as a scaffold to load bFGF for slow release into the vocal folds over the post-injection period as a preventative approach. From the viewpoint of wound repair, cells and biochemical events can be divided into the following stages: inflammatory reaction (inflammatory stage-a few days), cell proliferation and synthesis of elements which make up the extracellular matrix (proliferative stage ‒ 14 days), and the posterior period called remodeling (remodeling stage), which can last for one year or more.^22^ We hypothesized that treating injured vocal folds with a single prophylactic injection of bFGF with gelatin hydrogel microspheres as a drug delivery system immediately after creating an injury would interact with, and influence local vocal fold cells that modulate the wound environment, preventing the worsening of the vocal fold injury in the early phase of tissue repair, and might be leading to less scar formation. In order to assess the early effect of a prophylactic injection of gelatin hydrogel microspheres containing bFGF on tissue repair, we evaluated the vocal fold histology and mucosal movement for two weeks following injury creation as that period matches the abovementioned proliferative stage in which ｂFGF is supposed to improve wound healing effectively. The purpose of the current study was to develop a novel regenerative approach for the treatment of injured vocal folds after surgery, using biodegradable gelatin hydrogel microspheres as a drug delivery system for Basic Fibroblast Growth Factor (bFGF).

## Methods

This study was approved by the Institutional Review Board of Fukushima Medical University on November 30th, 2015 (Confirmation Number: #238), which is guided by local policy, national laws, and the World Medical Association Declaration of Helsinki.

### Preparation of injectable materials

Two types of injectable materials, gelatin hydrogel microspheres with bFGF and gelatin hydrogel microspheres without bFGF, were prepared as follows. Gelatin with an isoelectric point of 4.9 was isolated from bovine bone collagen using an alkaline process. By cross-linking with glutaraldehyde, the gelatin was prepared as a hydrogel and preserved in a freeze-dried form until use. The size of the microspheres ranged from 10 to 70 μm in the swollen state. We used a recombinant human bFGF (200 μg/mL) with an isoelectric point of 9.6, and applied this to the microspheres. The degradation time of the hydrogel was 14 days.^23^ bFGF was loaded in the microspheres for slow release into the vocal folds over the post-injection period.

### Surgical procedure

Animal care, housing, and surgical procedures were carried out in accordance with the guidelines of the Animal Experiment Committee at Fukushima Medical University.

A total of 16 Japanese white rabbits (male, 11 weeks old, body weight 2.0–2.4 kg) were purchased from Japan SLC, Inc. (Shizuoka, Japan). Two types of injectable materials were administered into 14 Japanese white rabbits. Two rabbits were placed into the non-injured vocal fold group as controls. The rabbits were anesthetized by an intramuscular administration of a cocktail of medetomidine hydrochloride (0.2 mg/kg; Nippon Zenyaku Kogyo Co, Ltd, Fukushima, Japan), midazolam (1.0 mg/kg; Astellas Pharma, Inc, Tokyo, Japan), and butorphanol tartrate (0.2 mg/kg; Meiji Seika Pharma Co, Ltd, Tokyo, Japan). The vocal folds were visualized using a steel mouth opener, and a 1.9 mm diameter, 0-degree endoscope (Olympus Co, Ltd, Tokyo, Japan) connected to an external light source and video monitor. A video documentation of the surgical procedures was accomplished. Each surgical procedure was performed by a surgeon with 14 years’ experience of performing human laryngeal surgery and eight years’ experience of conducting animal laryngeal surgery with an assistant. The depth and consistency of injury were confirmed by two observers experienced in laryngeal surgery.

**Figure 1:**
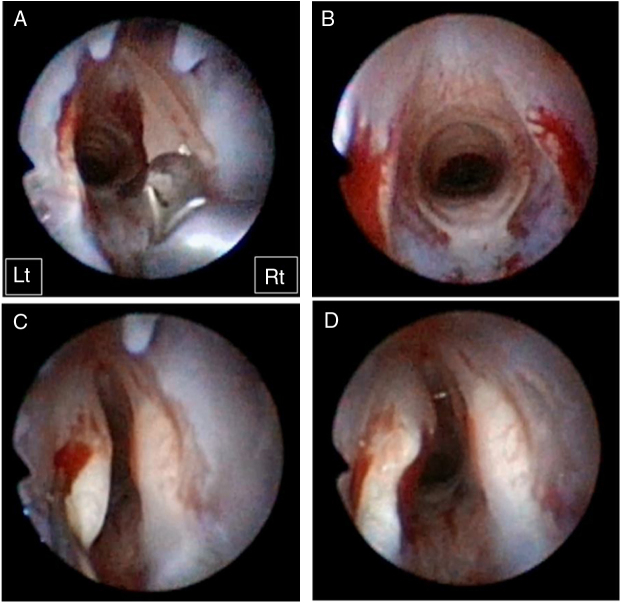
Surgical procedure. Bilateral vocal fold injury was created using microforceps (A). After confirmation of the bilateral consistent injury (B), a volume of 100 μL gelatin hydrogel microspheres without bFGF or gelatin hydrogel microspheres with bFGF was injected into the right or left injured vocal folds (C). Vocal fold bulging was observed during all injections (D). Rt and Lt indicate right side and left side, respectively.

Bilateral vocal fold injury was created by using micro-forceps to remove the mucosal and superficial layer of muscle (Fig. 1A). After confirmation of the injury consistency (Fig. 1B), either 100 μL of gelatin hydrogel microspheres without bFGF or the same microspheres with bFGF was randomly injected into the right or left injured vocal folds using a 22 gauge needle and a 1 mL syringe. To avoid gelatin hydrogel microsphere leakage, we allowed 4–5 min for gelation after mixing. We then injected the gelation material into the lamina propria (Fig. 1C). If the gelation time was prolonged, its completion would result in clogging of the needle or difficulty performing the injection. We observed vocal fold bulging during each injection (Fig. 1D). The maximum volume of injectable materials was tested as 100 μL without causing excessive tissue bulging that might compromise the airways of the rabbits. Two of the 14 rabbits were euthanized due to inconsistency of injury, which was evaluated by two observers. A decision of inconsistent injury requires discussion and agreement between said observers. A total of 12 rabbits (six rabbits in each treatment group [gelatin hydrogel microspheres without bFGF (non-bFGF group) and gelatin hydrogel microspheres with bFGF (bFGF group)] were used for data analysis. Two rabbits were placed into the non-injured vocal fold group without treatment as controls.

At day 14 post surgery, the rabbits were euthanized by intraperitoneal injection of pentobarbital sodium (Kyoritsu Seiyaku Corporation, Tokyo, Japan), and the larynges were excised for evaluation of vocal fold histology and mucosal movement.

### Histological examination

The resected larynges were fixed with 4% paraformaldehyde in phosphate buffered saline (pH 7.4) and embedded in paraffin. The larynges were sliced into 4 μm sections and subjected to Hematoxylin and Eosin (H&E) as well as Elastica Van Gieson (EVG) staining for light microscopic observation (BX-51; Olympus). H&E staining was used for morphological analysis; collagen content was visualized with EVG staining. Histological analysis was performed by a blinded independent pathologist.

### Vibratory examination of excised larynges and analysis of vocal fold vibration and shape by laryngologists

**Figure 2:**
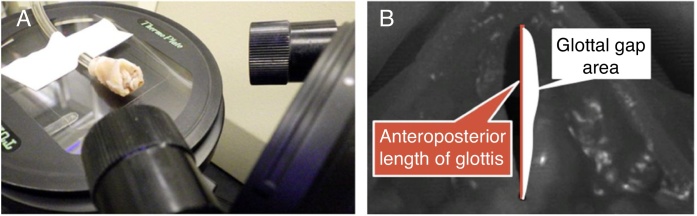
Vibratory examination. To record the vocal fold vibrations, a high-speed digital imaging system was mounted above the larynx and the images were recorded at a frame rate of 5000 frames/s (A). We calculated the index of vocal fold opening (IVFO), which is influenced by vocal fold scar formation. The following equation was used: IVFO = glottal gap area/anteroposterior length of glottis (B).

Vocal fold vibrations were examined with a high-speed digital imaging system before fixation with 4% paraformaldehyde in phosphate buffered saline. Vibratory examination was performed within 60 min of excision of the larynges to avoid the influence of rigor mortis. For visualization of the vocal fold mucosal movement, the epiglottis and false vocal folds were removed. Airflow (90 L/minute) was generated through a tube for the vibratory examination. To record the vocal fold vibrations, a high-speed digital imaging system (FASTCAM mini UX50; Photron, Tokyo, Japan) was mounted above the larynx, and the images were recorded at a frame rate of 5000 frames/s (Fig. 2A).

In this experiment, we calculated the Index of Vocal Fold Opening (IVFO) using the anteroposterior length of the glottis and the glottal gap area referring to previous studies.^21^, ^24^ IVFO is influenced by vocal fold scar formation and is thought to reflect functional recovery. Our pilot work showed that passive opening of the vocal fold generated by airflow depended on the severity of the scarring. Thus, the following equation was used: IVFO = glottal gap area/anteroposterior length of glottis (Fig. 2B).

Twelve injured vocal folds without treatment, six vocal folds of the non-bFGF group and six vocal folds of the bFGF group were examined. Each vocal fold opening was examined at the maximal abductive moment. The area of the glottal gap area and anteroposterior length of the glottis was measured using ImageJ software. Minor morphologic differences and asymmetry, which are generally difficult to distinguish through mechanical evaluation, were analyzed on vocal fold vibration videos by two senior blinded independent laryngologists: Evaluator 1 and Evaluator 2 are both specialists in laryngology with vast experience in phonosurgery and animal experimentation, respectively. Vocal fold vibration and shape were assessed using a five-grade scale referring to a stroboscopic assessment sheet from The Larynx 2^nd^ edition (Massachusetts Eye and Ear Infirmary Voice and Speech Laboratory). Each item was scored as: 1 = complete difference compared to normal vocal folds; 2 = severe difference compared to normal vocal folds; 3 = moderate difference compared to normal vocal folds; 4 = mild difference compared to normal vocal folds, and 5 = no difference compared to normal vocal folds.　　　　

### Statistical analysis

IVFO was compared between the injured vocal fold group and both the non-bFGF and bFGF groups using the Mann-Whitney *U*-test and post-hoc Bonferroni correction. Vocal fold vibration and shape were evaluated by two blinded independent laryngologists using the five-grade scale described in the Materials and Methods section. The average scores of vibration and shape were compared between the non-bFGF and bFGF groups using the Mann-Whitney *U*-test. All analyses were carried out using SPSS Statistics 23.0 software. A p-value of < 0.05 was considered statistically significant.

## Results

### Histological examination

**Figure 3:**
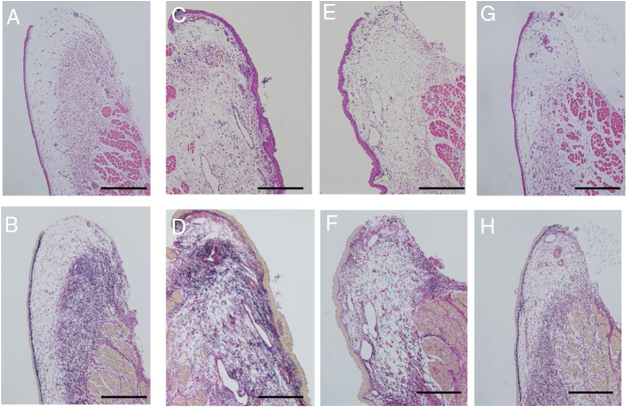
Histological examination. Sections were stained with Hematoxylin and Eosin (H&E), as well as Elastica van Gieson (EVG). Uninjured vocal folds were prepared as controls (A and B). The surface of the injured vocal folds is irregular with marked deformation covered by thick epithelium. Collagen fibers are diffusely distributed in the lamina propria (C and D). In the gelatin hydrogel microspheres injection without bFGF, the irregular elevation of mucosal surface with deformation was observed. The collagen density was slightly decreased relative to the injured vocal folds (E and F). Injection of gelatin hydrogel microspheres with bFGF recovered the normal structure of the vocal folds, which were composed of a flat surface and an orderly arranged collagen layer (G and H); (200 μm scale bar) (A, C, E and G were stained with H&E, and B, D, F and H with EVG).

Vocal fold injury was evaluated via assessment of the morphological deformation and density of the collagen fibers. Uninjured vocal folds were prepared as controls. The surface of the mucosa looked flat, and was covered by thin squamous epithelium. The collagen fibers were orderly arranged as a layer in the deep lamina propria (Figs. 3A and B). The damaged vocal folds showed an irregular and deformed surface with a thick epithelium. The collagen fibers were diffusely deposited in the whole layer of the mucosa (Figs. 3C and D), and the injured vocal folds of the non-bFGF group showed irregular elevation of the mucosal surface with deformation. The collagen density was slightly decreased relative to the injured vocal folds; however, the distribution of collagen fibers was not layered, unlike the non-injured mucosa. The epithelium was still thick compared to those of the uninjured vocal folds (Figs. 3E and F). The injured vocal folds of the bFGF group showed a flat surface with thin squamous epithelium. EVG staining exhibited layered deposition of the collagen fibers similar to that of the control mucosa (Figs. 3G and H). The histological findings showed homogeneity in all groups.

### Vibratory examination of the excised larynges

**Figure 4:**
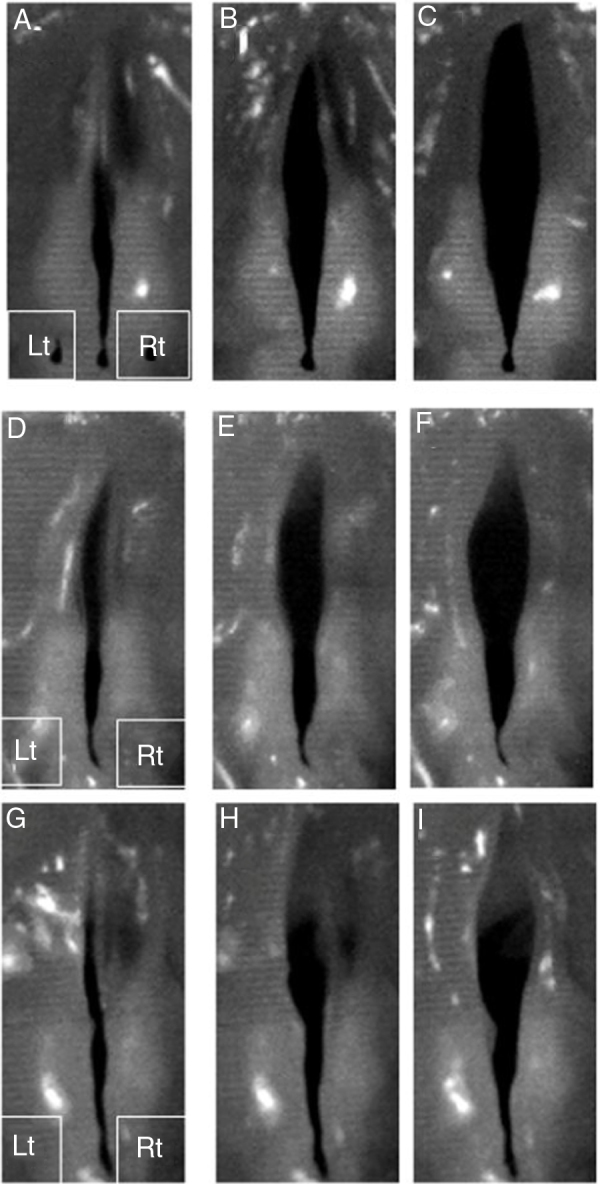
Vibratory examination of the excised larynges. Representative digital high-speed images demonstrated symmetric mucosal waves of non-injured vocal folds (A, B and C). All injured vocal folds shown on the left side revealed limited mucosal waves and vocal fold openings (D–I). The gelatin hydrogel microspheres without bFGF injected vocal folds seen on the right side demonstrated limited mucosal waves and vocal fold openings. The vibration and openings were better compared to injured vocal fold (D–F). The gelatin hydrogel microspheres with bFGF injected vocal fold on the right side revealed better vibration and vocal fold openings. The vibration and openings were still limited compared to the non-injured vocal fold (G–I). Rt and Lt indicate right side and left side, respectively.

Digital high-speed images demonstrated symmetric mucosal waves of non-injured vocal folds (Figs. 4A, B and C). All injured vocal folds showed limited mucosal waves and vocal fold openings compared to the non-injured vocal folds (Figs. 4D-I). The right vocal folds of the non-bFGF group had limited mucosal waves and vocal fold openings; however, the vibrations and openings were better compared to the injured left vocal fold without gelatin hydrogel microspheres injection (Figs. 4D-F). The right vocal folds of the bFGF group exhibited better vibrations and vocal fold openings; however, the vibrations and openings were limited compared to those of the non-injured vocal folds (Figs. 4 G–I).

**Figure 5:**
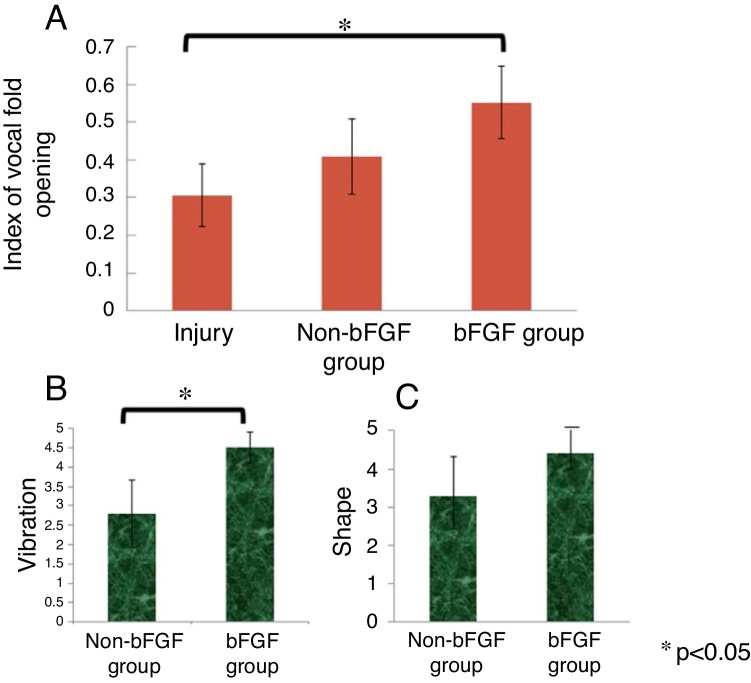
Digital analysis of IVFO. IVFO of the injured vocal folds, gelatin hydrogel microspheres without bFGF injected vocal folds and gelatin hydrogel microspheres with bFGF injected vocal folds were 0.31, 0.41 and 0.55, respectively. There were significant differences in IVFO between the injured vocal folds and gelatin hydrogel microspheres with bFGF injected vocal folds (A). The average vibration scores of the gelatin hydrogel microspheres without bFGF injected vocal folds and gelatin hydrogel microspheres with bFGF injected vocal folds were 2.8 and 4.5, respectively. There was a significant difference in average vibration score between the gelatin hydrogel microspheres without bFGF injected vocal folds and the gelatin hydrogel microspheres with bFGF injected vocal folds (B). The average shape scores of the gelatin hydrogel microspheres without bFGF injected vocal folds and the gelatin hydrogel microspheres with bFGF injected vocal folds were 3.3 and 4.4, respectively. The result was not statistically significant (C).

The average IVFOs of the non-injured vocal folds, injured vocal folds, gelatin hydrogel microspheres without bFGF-injected vocal folds, and gelatin hydrogel microspheres with bFGF injected vocal folds were 0.47, 0.31, 0.41 and 0.55, respectively. There was a significant difference in IVFO between the injured vocal folds and vocal folds of the bFGF group (*p* = 0.003). The non-bFGF group showed higher IVFO compared to that of the injured vocal fold group, and the bFGF group had higher IVFO compared to that of the non-bFGF group; however, the results were not statistically significant (*p* = 0.871 and *p* = 0.078, respectively) (Fig. 5A).

### Analysis of vocal fold vibration and shape by laryngologists

There were no significant differences in IVFO between the non-bFGF and bFGF groups. Two trained laryngologists using the previously described five-grade scale assessed the vocal fold vibration and shape. The inter-examiner difference for each measurement was less than one. The average vibration scores in the non-bFGF and bFGF groups were 2.8 and 4.5, respectively. There was a significant difference in average vibration score between the groups (*p* = 0.032) (Fig. 5B). The average shape scores in the non-bFGF and bFGF groups were 3.3 and 4.4, respectively. There was a higher average shape score in the bFGF group compared to that of the non-bFGF group, although the result was not statistically significant (*p* = 0.190) (Fig. 5C).

## Discussion

Regenerative potential of basic fibroblast growth factor contained in biodegradable gelatin hydrogel microspheres as a drug delivery system immediately applied following vocal fold injury was demonstrated by histological examination. Digital high-speed images of better mucosal waves also suggest a positive effect in injured vocal folds.

Two types of injectable materials, gelatin hydrogel microspheres without bFGF and gelatin hydrogel microspheres with bFGF, were tested in this study. A potent mitogen and chemoattractant for endothelial cells and fibroblasts, bFGF stimulates angiogenesis, metabolism, deposition of the extracellular matrix, and movement of mesodermally derived cells.^25^ Because of the favorable preventative effects of bFGF in the rat vocal fold injury model,^18^ we hypothesized that injecting gelatin hydrogel microspheres with bFGF immediately after creating the injury would lead to interaction with the local vocal fold cells that modulate the wound environment, providing prophylactic benefits for the injury. A number of studies have addressed the effectiveness of a combination of biodegradable gelatin hydrogel with controlled release of bFGF for the regeneration of damaged tissues or organs, including the skull bone,^26^ limbs,^27^ facial nerves,^28^ tympanic membrane^29^ and laryngeal nerves.^30^ No side effects due to the biodegradable gelatin hydrogel with bFGF have been observed in several previous studies, such as those focused on bone defects in rabbits,^26^ streptozotocin-induced diabetes in rats^31^ and skin ulcers in mice.^32^ There have been no reports on the use of injecting biodegradable gelatin hydrogel microspheres with bFGF into vocal folds immediately after surgical procedures. Kobayashi et al. reported the effects of using bFGF with gelatin hydrogel for restoring acute vocal fold scars. However, the regenerative effect on acute vocal fold scarring was limited.^21^ The reason for this might be that the injection was made into the scarred vocal fold one month after the surgery. Suzuki et al. reported that local application of bFGF has the potential to prevent vocal fold scarring in rat vocal folds; however, in that study, injection of saline with bFGF was performed before the surgical procedure to reduce the risk of leakage.^18^ Immediate injection of bFGF after surgery is technically challenging. Therefore, in the present study, gelatin hydrogel microspheres were selected in order to reduce leakage. The viscosity of gelation material is different from that of saline, which easily leaks from the injured area after injection. Gelation materials injected in a timely manner could reduce leakage from the injured vocal fold. According to the current study design, the treatment of injured vocal folds might contribute to the synergistic effects of bFGF on with gelatin hydrogel microspheres and resident cells in the vocal folds. However, further investigation might be warranted that includes an additional saline group with bFGF only, to characterize the effects of bFGF.

Our study results indicate exceptional effects of gelatin hydrogel microspheres with bFGF on injured vocal folds. However, the repaired vocal folds are not returned to normal, like uninjured vocal folds. Our results could be further improved by determining an adequate dose of bFGF. It has been reported that ischemic free-flap implantation increases survival rate in a dose-dependent manner.^33^ In the present study, injured vocal folds injected with a controlled release of 20 μg bFGF showed significantly less scar formation, demonstrating that the dose was adequate for treating injured vocal folds. Regarding bFGF application for vocal folds, Nagai et al. reported the effectiveness of 1 μg bFGF for rat vocal fold paralysis utilizing autologous fascia and gelatin hydrogel.^34^ In addition, Tamura et al. reported the effectiveness of 1 μg bFGF with fat tissue for human vocal fold augmentation to maintain fat tissue volume.^35^ However, another report has suggested that an inappropriate dose of bFGF produces granulation tissue.^30^ Komura et al. reported the effectiveness of intra-tracheal injection with a concentration of 200 μg/mL bFGF for tracheal cartilage growth, and no changes in breathing condition or formation of granulation tissue on histological examination were observed in any of the rabbits in their study.^36^ Because the trachea and larynx are continuous structures that form the airway, we determined a concentration of 200 μg/mL bFGF to be a safe concentration for intra-laryngeal injection for the prevention of suffocation caused by bFGF side effects. In the present study, two weeks of observation after vocal fold injection of gelatin hydrogel microspheres containing 200 μg/mL bFGF were accomplished safely without the formation of granulation tissue. To determine the most effective concentration of bFGF for treating injured vocal folds, dose-response studies are required.

We demonstrated significant differences in IVFO between injured vocal folds and gelatin hydrogel microspheres with bFGF injected vocal folds. No significant differences in IVFO between the non-bFGF and bFGF groups were observed. As previously reported, biocompatible hydrogels have the ability to modulate and enhance wound healing response in injured vocal folds.^37^ Due to tumor growth risk, bFGF is not always applicable for vocal fold surgery.^19^ Therefore, we hypothesized that if the effect of simply injecting gelatin hydrogel microspheres is comparable to that of injection of the same microspheres but with bFGF, the usage of the material would be facilitated more clinically. To prove this hypothesis, the non-bFGF and bFGF groups were additionally compared by a trained laryngologist using vocal fold vibration videos. The evaluations demonstrated that there was a significant difference in average vibration score between the non-bFGF and bFGF groups. The hypothesis that the effect of simple gelatin hydrogel microsphere injection is comparable with that of injection of gelatin hydrogel microspheres containing bFGF was not supported in the present study. Because of the identifiable differences in histological examination in injured vocal folds, the vibration of injured vocal folds was excluded from evaluation. However, evaluation of injured vocal fold vibration may provide useful information for future investigations to better quantify vocal fold scarring.

Limitation of this study was the lack of a long-term observation period after surgery. This is because our purpose was not to recover chronic vocal ford scarring, but to assess the early effects of a prophylactic injection of gelatin hydrogel microspheres containing bFGF on tissue repair. We evaluated the vocal fold histology and mucosal movement for two weeks following injury creation as that period matches the proliferative stage,^22^ and the tissue can be changed dramatically. In addition, as active inflammation in vocal fold injury is resolved in two weeks^5^ and the degradation time of the hydrogel was 14 days,^23^ we defined the observation period as two weeks. Previous studies on similar preventive effects of injectable hydrogel on vocal fold injury reported that treatment effects observed at Day 21 postoperatively were maintained during the chronic stage for 6 months.^6^, ^38^ However, long-term observation following surgery might be required to confirm changes in the characteristics of injured vocal folds in the chronic phase.

## Conclusions

A novel prophylactic injection of bFGF-containing biodegradable gelatin hydrogel microspheres as a drug delivery system demonstrates a regenerative potential for injured vocal folds after surgery in a rabbit model.

## Conflicts of interest

The authors declare no conflicts of interest.
